# Panic Attack Prediction for Patients With Panic Disorder via Machine Learning and Wearable Electrocardiography Monitoring: Model Development and Validation Study

**DOI:** 10.2196/69045

**Published:** 2025-10-15

**Authors:** Hayoung Oh, Hunmin Do, Chaehyun Maeng, Jinsuk Park, Taejun Yoon, Jihwan Kim, Hyeran Hwang, Seoin Choi, Piao Huilin

**Affiliations:** 1 Department of Artificial Intelligence Convergence, Sungkyunkwan University, 25-2, Seonggyun-gwan-ro, Jongno-gu, Seoul, 03063, Republic of Korea, 82 10-5389-5996; 2 Department of Mechanical Engineering, Sungkyunkwan University, Seoul, Republic of Korea; 3 Department of Child Psychology and Education, Sungkyunkwan University, Seoul, Republic of Korea; 4 Department of Computer Science and Engineering, Sungkyunkwan University, Seoul, Republic of Korea; 5 Department of Applied Data Science, Sungkyunkwan University, Seoul, Republic of Korea; 6 Department of Applied Artificial Intelligence, Sungkyunkwan University, Seoul, Republic of Korea; 7 School of Systems Biomedical Science, Soongsil University, Seoul, Republic of Korea

**Keywords:** electrocardiogram, heart rate variability, panic attack prediction mental health, wearable devices, digital mental health

## Abstract

**Background:**

Panic attack prediction remains a critical challenge in mental health care due to the high interindividual variability of physiological responses and the limitations of subjective psychological assessments.

**Objective:**

This study aims to develop a multimodal deep learning framework that integrates real-time physiological signals from wearable electrocardiogram (ECG) monitors and psychological assessments to improve the accuracy of panic attack prediction.

**Methods:**

We adapted the ConvNetQuake architecture, originally designed for seismic detection, to extract temporal patterns from ECG signals. The model was pretrained on the PTB-XL ECG dataset and fine-tuned using wearable ECG data collected from adult participants. In parallel, psychological profiles based on the *Diagnostic and Statistical Manual of Mental Disorders, 4th Edition* criteria and Panic Disorder Severity Scale assessments were encoded as auxiliary inputs. The multimodal framework was evaluated using standard performance metrics.

**Results:**

The proposed model achieved an accuracy of 71.43%, precision of 83.72%, recall of 70.59%, and F1 score of 76.60% in detecting heart rate variability anomalies associated with panic episodes. Experimental comparisons demonstrated that the integration of physiological and psychological modalities significantly outperformed unimodal baselines in prediction reliability.

**Conclusions:**

This study provides empirical support for wearable-based early warning systems for panic attacks. The proposed approach demonstrates the feasibility of just-in-time digital interventions and underscores the potential of wearable artificial intelligence in advancing affective computing and digital psychiatry.

## Introduction

### Advances in Electrocardiography Technology and Applications of Wearable Devices

Electrocardiography (ECG) is a test that records the heart’s electrical signals, invented in 1902 by Dutch physiologist Willem Einthoven. This invention has become a powerful tool for diagnosing various heart diseases and has significantly contributed to distinguishing between normal and pathological states [1]. The original electrocardiograph used a string galvanometer to record the voltage difference caused by the heart’s electrical activity across the limbs. However, due to a series of innovative discoveries and inventions by several key figures throughout the early 20th century, the 12-lead ECG system as we know it today was developed [2]. With the rapid advancement of information and communication technology and the widespread adoption of the Internet of Things, the number of portable devices has exponentially increased in recent years [3]. Additionally, an aging population has driven significant changes in the health care industry, focusing on the development of biosensors that enable real-time health monitoring. Among these devices, smartwatches are one of the most widely used wearable devices, capable of performing nearly all functions of a smartphone [3]. One such biosensor applicable to smartwatches is ECG, where these watches use a single electrode on the back of the watch face in contact with the wrist to record 1-lead ECG. Another electrode is placed on the front or side of the smartwatch, allowing users to activate the recording by touching the crown or surface with the opposite index finger (or hand). The list of smartwatches with ECG capabilities is continually expanding [4]. However, the ECGs captured by smartwatches still struggle to replace traditional 12-lead ECGs used clinically. This limitation is primarily due to critical issues like the uncertain tracings caused by noise in smartwatch recordings [5]. Nevertheless, recent studies demonstrate that smartwatches can detect certain levels of heart disease [6]. Additionally, with advancements in artificial intelligence, heart disease prediction algorithms integrated with artificial intelligence are continuously being proposed, suggesting that it may be possible to predict heart disease using single-lead or multilead data from smartwatches [7]. Current research indicates that a range of heart conditions can be detected and predicted through ECG-enabled smartwatches, with atrial fibrillation being the most extensively studied area [8].

### Association Between Mental Health and Cardiovascular Disease Through Heart Rate Variability Analysis

Stress-related mental health disorders, such as anxiety and depression, have emerged as independent risk factors for cardiovascular disease [9]. Anxiety and depression not only increase the risk of cardiovascular disease but also impact the disease’s progression and course, potentially worsening it. Patients with anxiety disorders generally exhibit a tendency toward reduced heart rate variability (HRV), indicating excessive sympathetic nervous system activation. HRV, which reflects the balance of the autonomic nervous system, serves as a critical indicator in understanding the relationship between anxiety disorders and other mental health issues. Studies suggest that a similar physiological mechanism may underlie the heightened cardiovascular risk in patients with anxiety disorder [10]. Likewise, patients with major depressive disorder also tend to show decreased HRV, which adversely affects cardiovascular health. Research indicates a close association between HRV and the severity of depression, with changes in HRV values before and after antidepressant treatment linked to symptom improvement [11]. This suggests that HRV may serve as a biological marker for depression. Furthermore, HRV can effectively distinguish specific mental health disorders, such as panic disorder, from generalized anxiety disorders. Existing research shows that machine learning models using HRV data can more accurately classify panic disorder and generalized anxiety disorder, demonstrating HRV’s vital role in identifying subtle differences between anxiety subtypes [12]. Consequently, HRV has potential as a tool for developing personalized treatment strategies for individual patients.

However, predicting panic attacks presents a far more complex challenge than predicting heart disease. Panic attacks are challenging to predict as they may occur unexpectedly and are influenced by a range of factors, including psychological state and environmental elements. Therefore, a predictive model that integrates not only ECG data but also stress levels, sleep patterns, and environmental factors is essential [13]. With advances in wearable technology allowing for real-time physiological data collection, the importance of providing early warning signals before the onset of a panic attack and establishing preventive management services has increased. This paper explores the potential and challenges of predicting panic disorder risk using wearable devices and HRV analysis, proposing strategies to address these challenges. By standardizing data preprocessing and combining psychological factors with physiological signals, a predictive model can provide users with early alerts about their panic disorder risk. This approach aims to enhance the quality of life for those showing symptoms of panic disorder and contribute significantly to preventive mental health management.

### Advantages and Potential of Real-Time ECG Data Collection via Wearable Devices

Real-time ECG monitoring using wearable devices presents an intriguing alternative to overcoming the limitations of 12-lead ECG tests. Wearable ECGs have consistently demonstrated reliable heart condition detection, such as arrhythmias, when compared to current standard treatments. Various studies emphasize that these devices have the potential to improve patient care and reduce health care costs [14]. Multiple wearable ECG devices are currently available on the market, many of which are certified by the U.S. Food and Drug Administration [15]. This suggests that ECG testing via wearable devices may have the potential to detect and predict heart disease at a clinical level. Wearable ECGs are particularly useful for continuously monitoring cardiovascular conditions in patients with chronic illnesses, as they provide superior accessibility by enabling monitoring outside of hospital settings.

Additionally, continuous data collection allows physicians to tailor effective treatments to the specific characteristics of individual patients [16]. Although data limitations, noise, and reliability issues currently hinder wearable ECGs from fully replacing 12-lead ECG tests, the accumulation of more data, integration of artificial intelligence, and advancements in noise reduction techniques could make wearable ECGs a practical and highly accurate solution for patients with heart disease in the future. Meanwhile, data collection through wearable devices also plays a significant role in predicting mental health issues such as panic attacks. Wearable devices are particularly advantageous for the real-time monitoring of physiological signals, including ECG and HRV. Studies related to panic attacks have shown that data from wearable devices—such as heart rate, sleep stages (deep sleep, light sleep, and rapid eye movement (REM) sleep), and physical activity—are critical in predicting panic attacks, enabling long-term tracking of an individual’s physiological state and daily variations for more precise prediction [13]. Physiological indicators such as HRV are also useful for detecting mental fatigue or anxiety. Fatigue states have been detected with over 75% accuracy using HRV data from wearable devices, with the k-nearest neighbors algorithm yielding the highest performance [17]. Thus, the collection of physiological data using wearable devices demonstrates potential not only for the early detection of mental health issues such as panic attacks but also for detecting psychological factors such as fatigue and anxiety. In conclusion, data collected through wearable devices play a crucial role in the real-time detection of mental health issues, including panic attacks, fatigue, and anxiety. Furthermore, long-term and consistent data collection enables wearable devices to become an innovative approach to mental health management [18].

### Effectiveness of Single-Lead-Based Models for Detecting Panic Disorder and Panic Attacks

Recent studies highlight the potential of machine learning models based on ECG and HRV data for predicting panic attacks. Panic attacks, characterized by sudden physical and psychological symptoms, present a complex issue, and their early prediction and intervention can significantly improve patients’ quality of life. Studies utilizing multiple physiological signals have applied various algorithms, such as logistic regression, k-nearest neighbors, support vector machine, random forest (RF), federated learning, and multilayer perceptron, to distinguish patients with panic disorder from healthy controls. Multilayer perceptron, in particular, recorded a predictive accuracy of 75.61%, with ECG and peripheral temperature features derived from stress and recovery stages acting as key predictive indicators [19]. This demonstrates that multisignal analysis based on ECG and HRV data can maximize the effectiveness of panic attack prediction.

However, it has been revealed that physiological signals alone are limited in further improving the accuracy of panic attack prediction. Consequently, recent studies have developed predictive models that integrate psychological factors alongside physiological signals. Research employing deep learning algorithms, such as recurrent neural networks and long short-term memory networks, has shown that initial psychiatric assessments—such as Beck Depression Inventory, Beck Anxiety Inventory, and State-Trait Anxiety Inventory—serve as more significant predictive factors than physiological data.

These models, combining physiological signals and psychological data, have raised prediction accuracy for panic attacks from 75.6% to 92.8%. Additionally, sleep duration has proven to be a critical protective factor, with sleep times between 6 hours and 23 minutes and 10 hours and 50 minutes showing a positive effect in preventing panic attacks [20]. Another study focuses on predicting panic attacks by integrating not only physiological and psychological signals but also environmental factors. Recent research demonstrates that a multifactor predictive model, including environmental data such as the air quality index, can enable seven-day predictions for panic attacks using machine learning. Specifically, a model using the random forest algorithm achieved a high prediction accuracy of 81.3%, with models that integrate physiological signals, psychological factors, and environmental data performing better than models using only a single data source [13].

## Methods

### Data Collection and Preprocessing Strategy

Understanding the biomarkers of panic attacks can improve diagnosis, treatment, and prevention. ECG data offer crucial insights into the cardiovascular characteristics associated with these intense anxiety episodes. This study proposes ECG data collection and preprocessing strategies specifically tailored to panic attack research, presenting a goal-oriented approach that goes beyond existing datasets.

### Data Collection

Raw ECG signals were retrieved from the large-scale multi-institutional dataset provided by Shaoxing People’s Hospital and Ningbo First Hospital [21] to enhance model generalizability. Although this study did not include external validation using a completely independent dataset, the use of multi-institutional data contributes to partial external validity. However, the lack of an external validation dataset remains a limitation. The dataset was selected in part because its ECG sampling rate of 500 Hz matches that of the Samsung Galaxy Watch 6, making it suitable for developing and validating smartwatch-compatible ECG analysis algorithms.

### Feature Extraction

ECG processing was carried out using the ecg.ecg() function from the BioSPPy Python library [22], which applies various signal processing techniques to identify key features of the ECG waveform, particularly R-peaks. As the most prominent peak in the ECG cycle, the R-peak serves as the basis for calculating important features such as HRV.

### HRV Calculation

HRV was calculated using the root mean square of successive differences (RMSSD) method based on R-peaks. This method calculates the time between successive R-peaks (RR intervals), determines their differences, and computes the square root of the mean squared differences, providing a reliable indicator of short-term HRV that reflects autonomic nervous system function. In addition to HRV, the BioSPPy library calculates the average RR interval to derive heart rate (beats per minute), while the detected R-peak count gauges heart rhythm and regularity over the analyzed period. Graphical visualizations with marked R-peaks are also provided to enable rapid evaluation of signal quality and detection accuracy.

### Feature Labeling: Probability Labeling Approach

Target values were derived using probability calculations based on age-specific normal RMSSD ranges [23]. An age-stratified range database containing unique median and range values for each age group was constructed. For instance, young adults aged 20‐29 years exhibit a median range of 0.0041‐0.0048 seconds with a total range of 0.0013‐0.0161 seconds, while older adults aged 60‐69 years show a median range of 0.00204‐0.000207 seconds and a total range of 0.0005‐0.0104 seconds, reflecting increased variability with age. RMSSD values were converted to milliseconds, and standardized scores were calculated based on range width and median values, assuming a normal distribution encompassing 96% of the data. The within-range values were probabilities calculated using the cumulative distribution function, whereas the out-of-range values were assigned probabilities based on an exponential decay function relative to the distance from the boundary. The study follows the “Guidelines for Developing and Reporting Machine Learning Predictive Models in Biomedical Research” [24].

### Ethical Considerations

The datasets used in this study (Shaoxing People’s Hospital and Ningbo First Hospital) were publicly available and fully anonymized; therefore, Institutional Review Board (IRB) approval and informed consent were not required for the retrospective analyses. An IRB application for a prospective user study involving patients with panic disorder and healthy controls has been submitted and is currently under review. All data used in this study were anonymized prior to access to ensure participant privacy. No personally identifiable information was available to the investigators. As this study did not involve direct recruitment, no participant compensation was provided.

## Results

### Limitations and Challenges of Transformer-Based Deep Learning Models

ECG signals exhibit complex temporal and spatial patterns, requiring diverse analytical approaches. ECG data encompass both immediate features (eg, HRV) and long-term heart rate patterns, necessitating an appropriately designed model for comprehensive analysis. In this study, various machine learning models and the deep learning-based transformer model were employed to analyze ECG signals.

**Figure 1.**
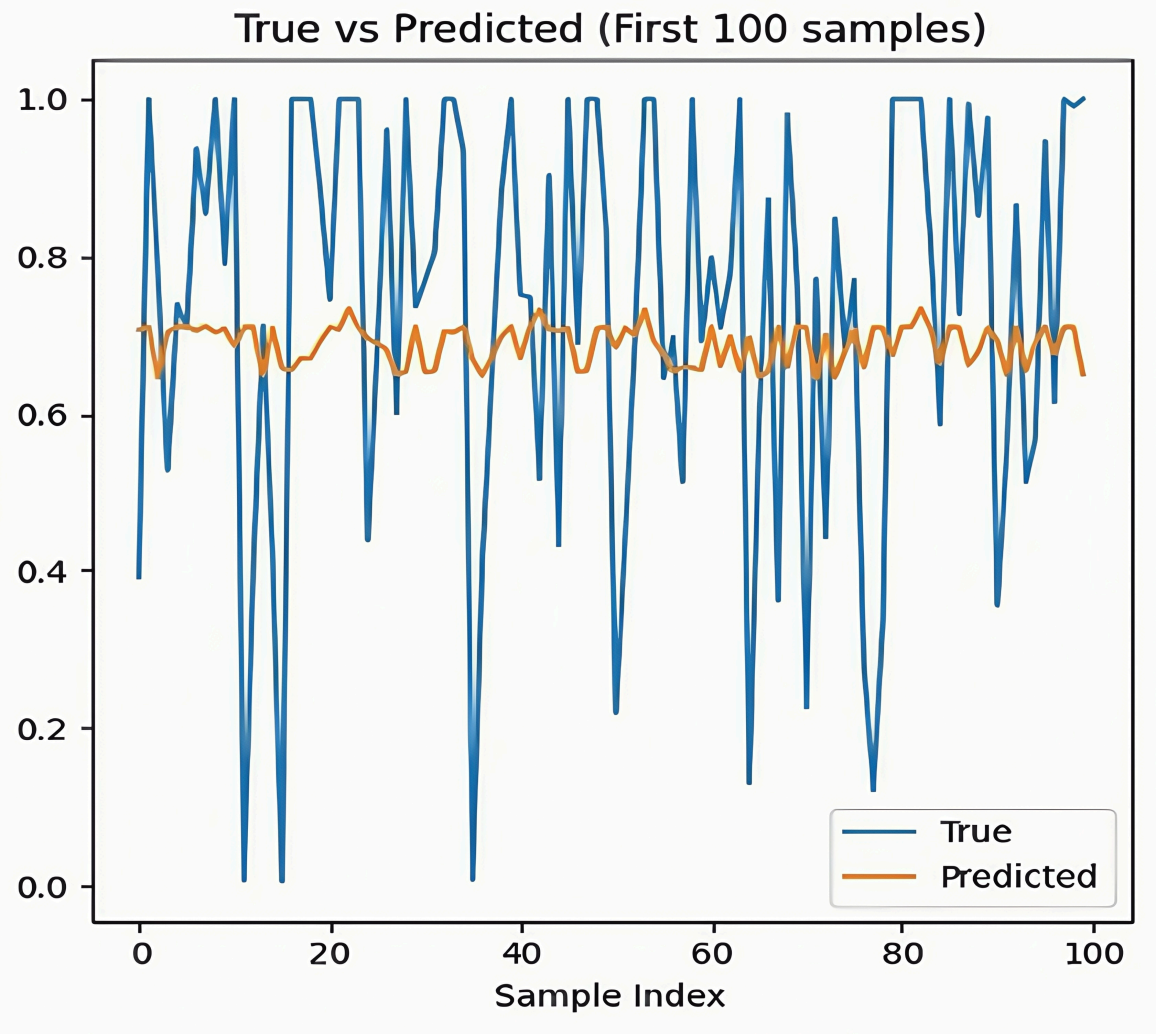
Prediction output of the transformer model for a single test sample visualization of the model’s predicted values compared with actual labels for the first 100 samples in the test set, based on 24-hour electrocardiogram (ECG)-derived heart rate variability (HRV) data collected from adult participants at Shaoxing People’s Hospital and Ningbo First Hospital (2021‐2023). The prediction distribution is flattened, indicating the transformer’s limited ability to differentiate between normal and abnormal patterns due to class imbalance. This figure highlights the need for improved modeling strategies or data augmentation techniques to address label sparsity in panic attack prediction tasks.

The analysis of ECG data using a transformer-based deep learning model revealed several limitations. Initial analysis of the prediction score distributions revealed no distinct boundary between the two classes under investigation (Figure 1). For clarity in subsequent analyses, we will denote these classes as Negative (previously Normal) and Positive (previously Abnormal), following standard binary classification terminology. Most prediction scores tended to cluster around a specific range (approximately near 0.6), which appears to stem from limitations in the training data and model architecture. Regarding the training data, the class imbalance is likely a major cause. If the amount of Positive Class data is significantly smaller than that of the Negative Class or if the data are skewed toward specific patterns, the model may struggle to learn the differences between the two classes. As a result, the model may fail to generalize effectively, leading to prediction outcomes being concentrated within a narrow range.

The limitations of the model architecture also warrant attention. ECG signals exhibit strong temporal dependencies and nonlinear patterns, and the transformer model may have been unable to sufficiently learn these characteristics. Specifically, the attention mechanism might have struggled to effectively capture subtle changes in the signal, such as R-peaks, which are critical features of ECG data. Additionally, the model may have overfit to noise present in the data or missed key features, suggesting issues with the model’s learning efficiency.

**Figure 2.**
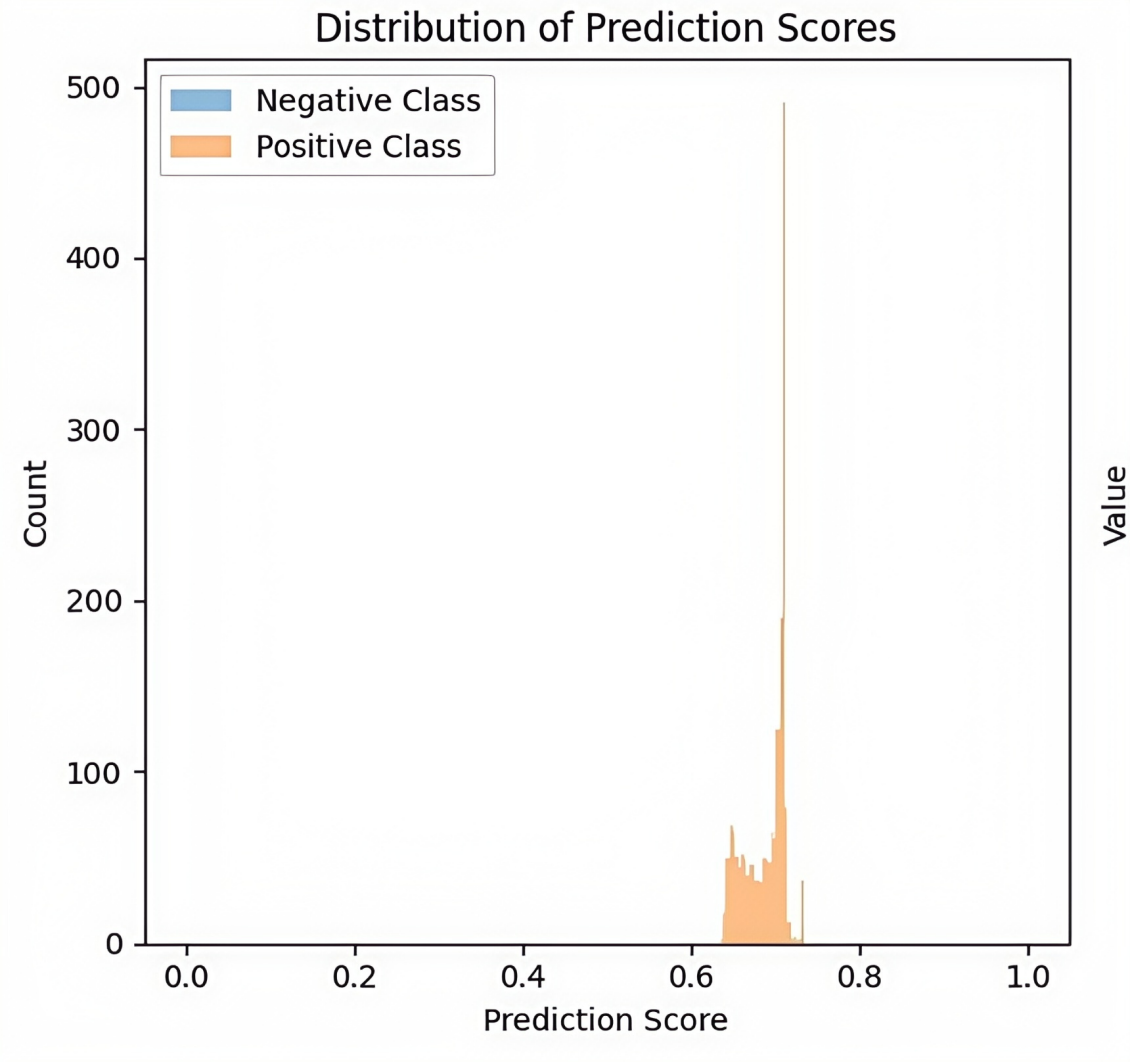
Distribution of prediction scores generated by the transformer model. A histogram visualizes the predicted probabilities for each class label across the test set using a transformer model trained on 24-hour electrocardiogram (ECG)-derived heart rate variability (HRV) data from adult participants at Shaoxing People’s Hospital and Ningbo First Hospital (2021‐2023). We initially hypothesized that a transformer-based architecture would be well suited for capturing the complex temporal dependencies inherent in ECG signals, especially given its self-attention mechanism’s ability to model long-range sequential patterns. However, the model failed to learn meaningful class separations, yielding an AUC of 0.50. The histogram shows a concentration of predicted scores around 0.6, with minimal separation between negative and positive classes. This suggests suboptimal feature encoding and ineffective decision boundaries, potentially caused by class imbalance, low temporal resolution in HRV sampling, and insufficient training data for deep sequential models.

Secondly, a comparison between true values and predicted values revealed that the model failed to adequately reflect changes in the actual data. Analyzing the first 100 data points from the test samples showed that the predicted values fluctuated within a limited range and did not accurately follow the abrupt changes in the actual values. This suggests that the model tended to output average-like values rather than effectively learning the key features of the data (Figure 2).

**Figure 3.**
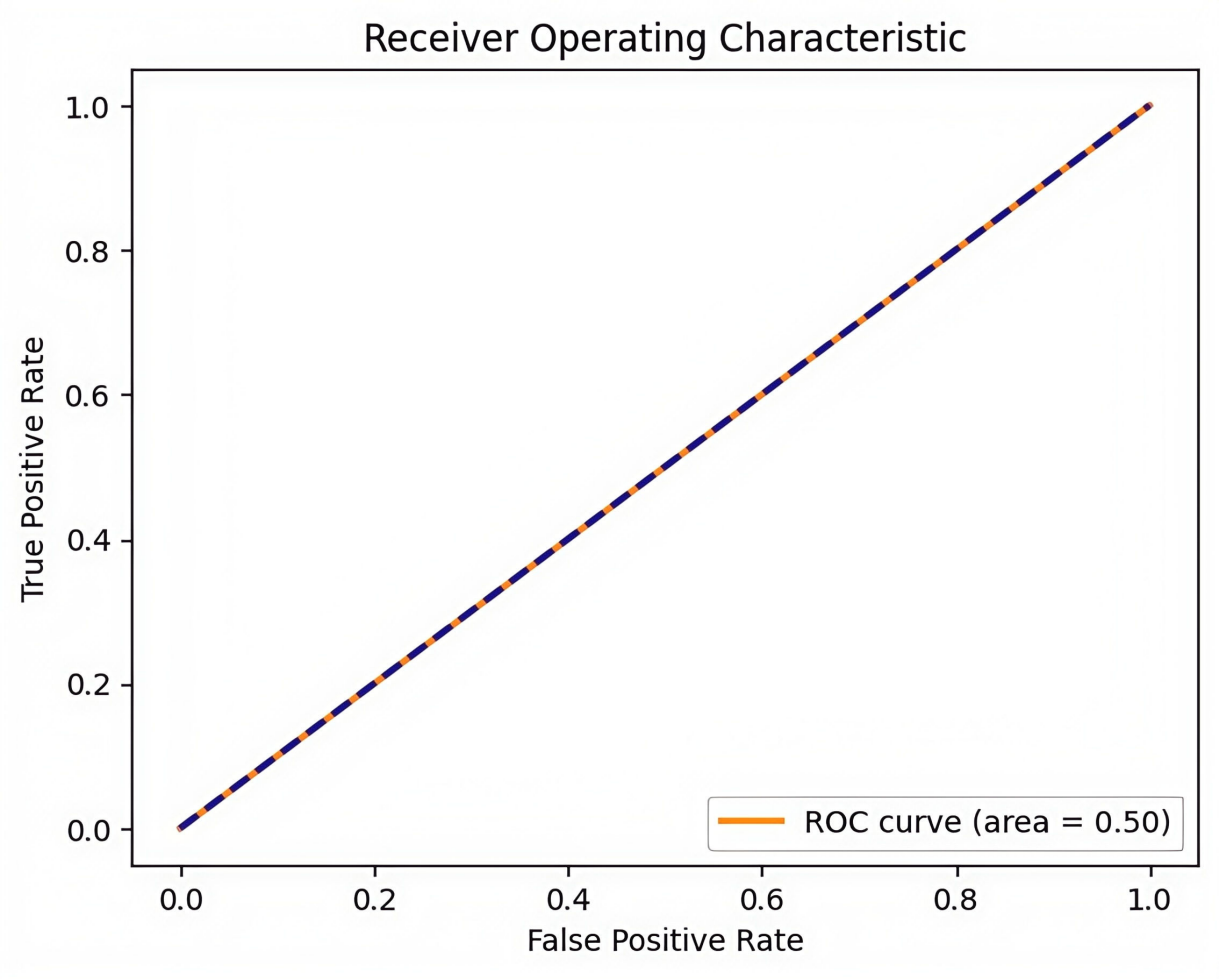
Receiver operating characteristic (ROC) curve of the transformer model ROC curve generated by the transformer-based classifier, based on electrocardiogram (ECG)-derived heart rate variability (HRV) data collected from adult participants at Shaoxing People’s Hospital and Ningbo First Hospital (2021‐2023). The model achieved an AUC of 0.50, indicating performance at random chance level. This outcome reflects the model’s inability to extract meaningful signal features from the physiological data, possibly due to class imbalance, insufficient temporal sensitivity, or limitations in the feature extraction process.

Thirdly, the ROC-AUC analysis result was 0.5, indicating that the model’s predictive performance was at the level of random guessing. ROC-AUC is a key metric for evaluating the performance of predictive models, and this result demonstrates that the transformer model failed to learn the temporal dependencies and subtle pattern changes in ECG data (Figure 3). This suggests the possibility that critical features were lost during the data preprocessing stage, in addition to the structural limitations of the model.

### Optimizing ECG Analysis Performance With Machine Learning Models

After identifying these issues, it was concluded that a transformer-based approach alone is insufficient to achieve satisfactory results in predicting cardiovascular abnormalities or panic attacks using ECG data. To overcome the limitations of the existing model and improve performance, additional experiments were conducted using machine learning models. Machine learning models offer a simpler structure and higher interpretability, enabling more effective learning of key data features. In particular, various algorithms were applied to address the data learning and classification performance limitations observed in the deep learning model. Following the deep learning model analysis, the performance of machine learning models in processing and classifying ECG data was further evaluated. Machine learning models, with their relatively simple structures and high interpretability, provide an opportunity to explore suitable approaches for ECG signal analysis through performance comparisons with deep learning models. Such comparisons yield critical insights into the strengths and limitations of each methodology, ultimately aiming to optimize ECG signal analysis.

This research presents an in-depth analysis of machine learning approaches to ECG data, leveraging a substantial dataset of 451,524 ECG recordings from the Shaoxing and Ninbo ECG database. The study focused on developing and optimizing models to assess HRV through RMSSD analysis, incorporating age-specific considerations and clinical relevance.

The dataset’s foundation comprised six key features, with demographic and physiological measurements including age (mean 58.0, SD 13.98 years), sex (male or female), RMSSD (mean 0.0789, SD 0.1411), mean heart rate (mean 50.8, SD 15.42 beats per minute), and R-peak count (mean 12.64, SD 37.62). The target variable was a probability measure between 0 and 1, indicating how typical an individual’s RMSSD measurement is for their age group as previously explained.

**Figure 4.**
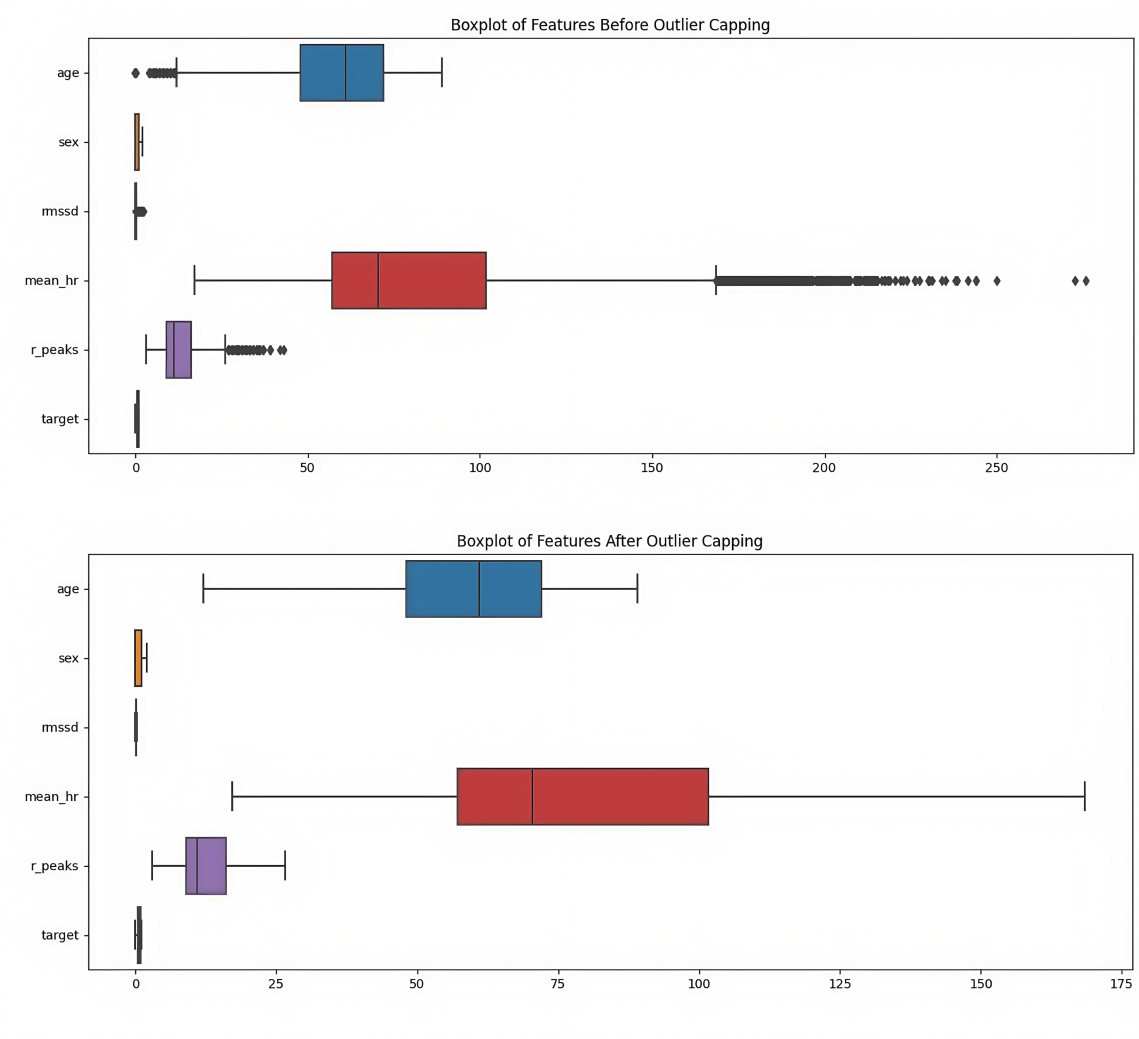
Boxplot of physiological features before and after clipping using interquartile range (IQR) from electrocardiogram (ECG) data collected across two hospitals (Shaoxing and Ningbo, 2021‐2023). Side-by-side boxplots show the distribution of key physiological variables—age, heart rate (HR), root mean square of successive differences (RMSSD), and R-peak count—before and after outlier clipping using the IQR method. Data were collected from adult participants using wearable ECG monitors at Shaoxing People’s Hospital and Ningbo First Hospital between 2021 and 2023. This comparison illustrates the impact of IQR-based preprocessing on improving feature reliability by mitigating extreme values.

**Figure 5.**
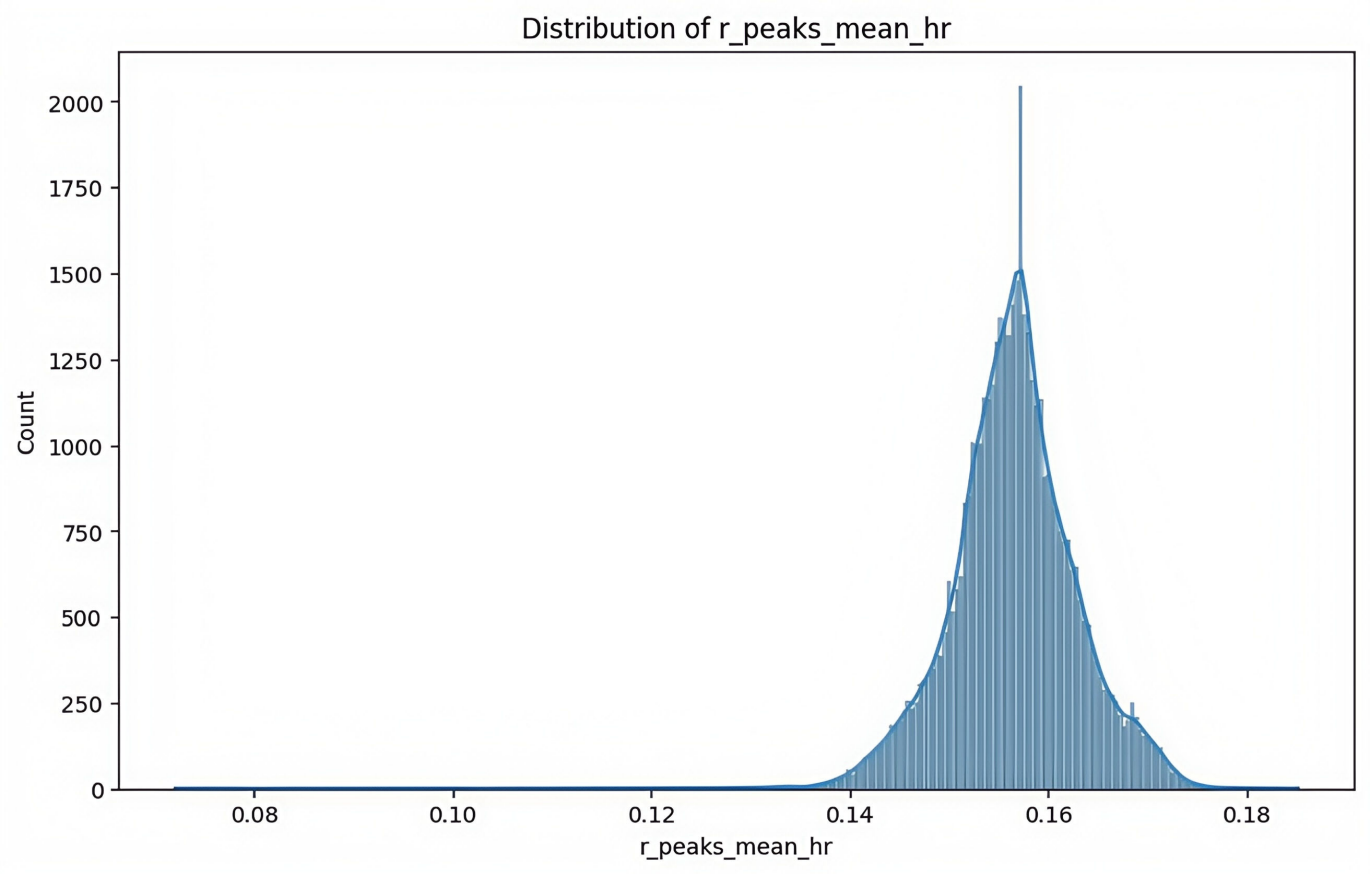
Distribution of the synthetic feature combining R-peak count and heart rate from electrocardiogram (ECG) data collected across two hospitals (Shaoxing and Ningbo, 2021‐2023). A histogram illustrates the distribution of the engineered feature (r_peak_mean_hr), created to mitigate multicollinearity between R-peak count and mean heart rate. Data were obtained from wearable ECG monitors used by adult participants at Shaoxing People’s Hospital and Ningbo First Hospital. The normalized bell-shaped distribution enhances model interpretability and feature robustness in downstream machine learning applications.

**Figure 6.**
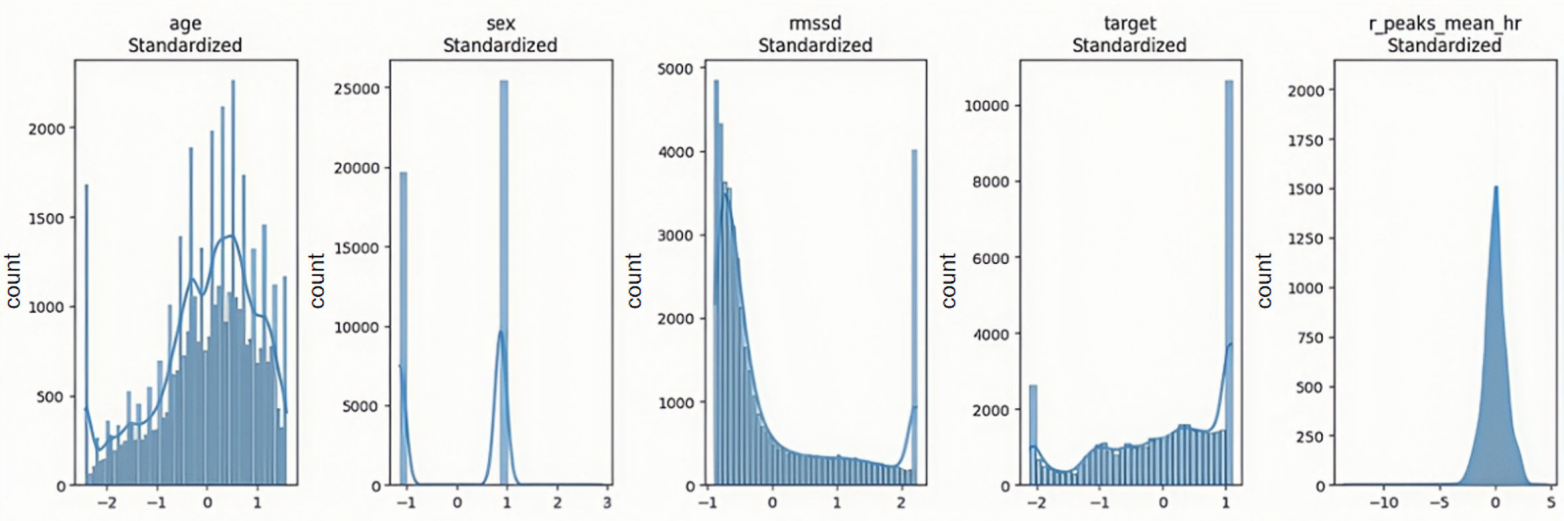
Standardized feature distributions after normalization from wearable electrocardiogram (ECG) data collected in two hospitals (Shaoxing & Ningbo, 2021‐2023). Histograms show the distribution of physiological features (age, sex, root mean square of successive difference [RMSSD], target label, r_peak_mean_hr) after applying StandardScaler (mean=0, variance=1). The data were obtained from adult participants using wearable ECG monitors at Shaoxing People’s Hospital and Ningbo First Hospital. This normalization process ensures that features contribute equally during model training, preventing scale-related bias.

The data preprocessing pipeline implemented a comprehensive approach to ensure data quality and model reliability. Outlier treatment employed the interquartile range method, with values capped at the 5th and 95th percentiles to maintain data integrity while preserving meaningful variations (Figure 4). A critical aspect of the preprocessing involved addressing multicollinearity through Spearman rank correlation analysis, which identified significant relationships between R_peaks and mean heart rate (*P*>.85; Table 1). This led to the development of synthetic features that effectively captured physiological relationships while reducing redundancy in the feature space (Figure 5). Feature normalization was achieved through the StandardScaler application, ensuring all variables contributed proportionally to the model learning process by transforming features to have zero mean and unit variance (Figure 6).

**Table 1.** Spearman’s rank correlation matrix of physiological features from wearable electrocardiogram (ECG) data collected at Shaoxing People’s Hospital and Ningbo First Hospital (2021-2023)^a^.

	age	sex	rmssd	mean_hr	r_peaks
age	1	0.0681	0.2096	0.03151	0.02895
sex	0.0681	1	0.0456	0.1084	0.105
rmssd	0.2096	0.0457	1	0.1454	0.1459
mean_hr	0.0315	0.1084	0.1454	1	0.9838
r_peaks	0.0289	0.105	0.1459	0.9839	1

a Pairwise correlation coefficients among input variables. A strong positive correlation between mean heart rate and R-peak count (ρ=0.9838) was observed, which informed the creation of a synthetic feature to reduce redundancy.

**Figure 7.**
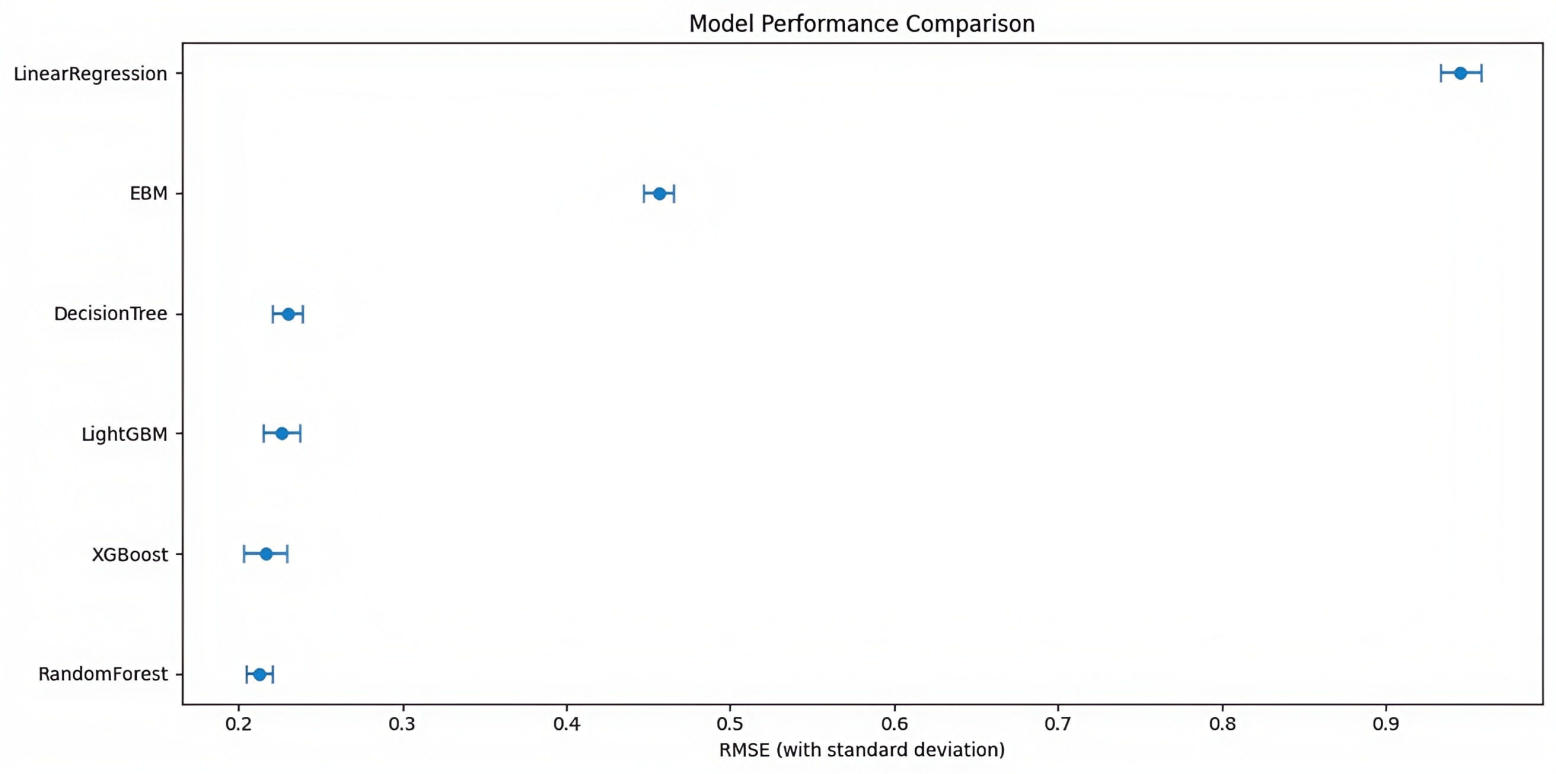
Model performance comparison by mean squared error (MSE) across six machine learning models trained on physiological features collected from wearable electrocardiogram (ECG) data (Shaoxing and Ningbo, 2021‐2023). Comparison of MSE with standard deviation across regression models: linear regression, decision tree, XGBoost, LightGBM, explainable boosting machine (EBM), and random forest. Models were trained using data collected from adult participants monitored over 24 hours via wearable ECG sensors. Among all models, random forest achieved the lowest MSE, demonstrating superior performance in predicting physiological anomalies linked to panic attacks.

The model development phase began with a systematic exploration of various regression algorithms, including linear regression for baseline linear relationship assessment, decision tree regression for nonlinear pattern capture, and advanced ensemble methods such as XGBoost and LightGBM. The RF model emerged as the superior approach, achieving performance metrics with an *R*² score of 0.954804 and root mean squared error of 0.2125 (Figure 7). The model’s architecture underwent optimization through Bayesian techniques, resulting in 111 decision trees with a maximum depth of 26 levels, effectively balancing model complexity with generalization capability.

**Figure 8.**
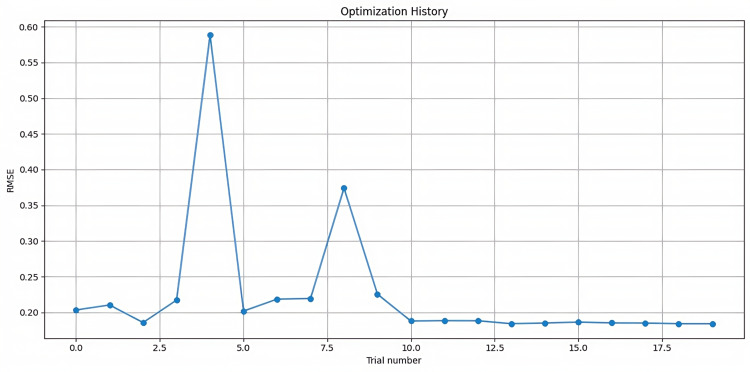
Hyperparameter optimization history of the random forest model on electrocardiogram (ECG) data from Shaoxing and Ningbo hospitals (2021‐2023). Line plot shows iterative improvement of the random forest model’s performance during hyperparameter tuning using a Gaussian optimizer. Data were derived from wearable ECG monitoring conducted across Shaoxing People’s Hospital and Ningbo First Hospital between 2021 and 2023. The graph illustrates convergence behavior toward the optimal parameter set by tracking mean squared error (MSE) across trials.

Model robustness was enhanced through bootstrap sampling and feature bagging, while critical hyperparameters underwent fine-tuning using cross-validated performance metrics. The optimization process used a Gaussian model, demonstrating superior efficiency compared with a traditional grid or random search methods (Figure 8). Cross-validation results showed exceptional stability with a mean squared error of 0.1369, variance of 0.0037, and an F1 score of 99.09%.

As shown in Table 2, feature importance analysis through the inherent ranking capability revealed that RMSSD and age were the most significant predictors [25], validating the model’s alignment with clinical knowledge. The final architecture demonstrated an exceptional ability to handle nonlinear relationships while maintaining interpretability. The ensemble nature provided natural uncertainty estimates through tree variance, offering valuable confidence metrics for clinical decision support [26]. The implementation included efficient processing pipelines for both batch and streaming data scenarios, with a modular design ensuring adaptability to various clinical requirements while maintaining consistent performance.

**Table 2.** Relative importance of input features in the random forest model trained on wearable electrocardiogram (ECG) data from adult participants (Shaoxing and Ningbo, 2021-2023)^a^.

Feature	Importance score	Interpretation
RMSSD^b^	0.79	Most significant predictor; aligns with clinical knowledge of autonomic dysfunction
Age	0.18	Second strongest predictor; consistent with age-related HRV^c^ decline
r_peak_mean_hr	0.02	Minor contribution; synthetic feature to reduce redundancy
Sex	0.01	Minimal contribution to prediction performance

a This table shows the normalized importance scores of each feature and their clinical interpretation. RMSSD and age were identified as the most influential features, validating the model’s alignment with clinical knowledge.

b RMSSD: root mean square of successive difference.

c HRV: heart rate variability.

This comprehensive approach resulted in a robust, clinically relevant model that effectively balanced accuracy and interpretability.

Comparative analysis of ensemble models showed strong performance across the board, with LightGBM achieving 98.5% classification accuracy (97.95% precision, 98.31% recall, and F1 score: 98.13%) and XGBoost demonstrating similar capabilities (98.44% accuracy, 98.53% precision, 97.56% recall, and F1 score: 98.04%). Feature importance analysis through the inherent ranking ability of RF confirmed RMSSD and age as the most significant predictors, validating the model’s alignment with clinical understanding.

The final implementation was able to handle nonlinear relationships while maintaining interpretability, with the ensemble nature providing natural uncertainty estimates through tree variance. The model’s design incorporated efficient processing pipelines for both batch and streaming data scenarios, ensuring adaptability to various clinical requirements while maintaining consistent performance. This comprehensive approach resulted in a robust, clinically relevant model that effectively balances accuracy and interpretability, suggesting significant potential for practical implementation in health care settings.

## Discussion

### Principal Findings

Panic attacks can be predicted using machine learning and deep learning models based on ECG signals and HRV data. In this study, the random forest algorithm achieved 99.27% accuracy on the test set, with 99.69% precision, 98.49% recall, and an F1 score of 99.09% using 45,152 ECG records, demonstrating the highest performance in detecting abnormal HRV (Table 3). While the transformer-based deep learning model revealed limitations in handling class imbalance and temporal dependencies, the machine learning model overcame these challenges and provided more interpretable prediction results.

**Table 3.** Classification performance metrics of machine learning models trained on wearable electrocardiogram (ECG) data from Shaoxing and Ningbo hospitals (2021‐2023)^a^.

	Accuracy	Precision	Recall	F1 score	RMSE^b^
Random forest	0.9927	0.9969	0.9849	0.9909	0.1369
XGBoost	0.9844	0.9853	0.9756	0.9804	0.1517
LightGBM	0.985	0.9795	0.9831	0.9813	0.1427
Federated learning	0.9176	0.9356	0.9411	0.9487	0.1972

a Summary of classification metrics (accuracy, precision, recall, F1-score, and RMSE) for four models—random forest, XGBoost, LightGBM, and Federated Learning—evaluated on test data derived from ECG monitoring of adult patients across Shaoxing People’s Hospital and Ningbo First Hospital between 2021 and 2023. Random forest outperformed the other models, achieving the highest accuracy (99.27%).

b RMSE: root mean squared error.

Key features for panic attack prediction were derived from physiological data, including RMSSD-based HRV calculations, mean heart rate, and R-peak counts, combined with age-specific variability in the data preprocessing strategy. The integrated model, which utilized all available data, outperformed the deep learning model and established itself as the optimal framework for ECG signal analysis.

This study highlights the potential of wearable devices for real-time ECG monitoring as a critical tool for early warning and prevention of panic attacks. It proposes significant possibilities for enhancing early intervention and preventive management strategies in mental health care.

### Comparison With Prior Work

A previous study has explored predicting panic attacks one week in advance by integrating wearable device data with survey-based inputs [13]. The study utilized survey-based labeling methods that included heart rate, sleep data, and environmental data, optimized using a random forest model. However, this approach had several limitations, such as recall bias in the survey-based labeling process and accuracy issues with environmental data. Additionally, the use of physiological data was restricted to heart rate, and the model’s applicability was limited to patients diagnosed with panic disorder.

In addition to Tsai et al [13], our approach differs from Gu and Hu [27] who focused on deep learning models using wearable mood monitoring. Unlike their approach, we applied a probabilistic age-adjusted HRV normalization to prevent overfitting to age-biased HRV thresholds and incorporated federated learning for privacy. In our federated setup, model parameters are trained locally on devices and aggregated centrally, ensuring that sensitive ECG data never leave the source institution.

In contrast, this study precisely analyzed abnormal heart rate patterns based on ECG signals and HRV data, effectively learning temporal dependencies and frequency-based features using a random forest model. Furthermore, systematic preprocessing methods, including data normalization, synthetic feature generation, and multicollinearity resolution, significantly improved data reliability and model performance. This approach demonstrates potential not only for early warning of panic attacks but also for analyzing patterns in other mental health issues such as post-traumatic stress disorder and anxiety disorders. To promote transparency and reproducibility, we provide information on data and code availability.

### Future Work

#### Addressing Clinical Applicability Through Real-World Data

In this study, we predicted panic attacks using 45,152 records from the Shaoxing and Ningbo ECG database. However, as this research involved predicting RMSSD using existing ECG datasets, it has the limitation of lacking data tested in real clinical settings. Therefore, as part of the future direction of this study and to enhance clinical applicability, we have submitted an application to the IIRB for a study involving both patients with panic disorder and the general population, which is currently under review.

Upon completion of the IRB review, we plan to measure ECG data from 10 patients with panic disorder and at least 20 individuals from the general population. This approach is expected to improve the reliability of the model and facilitate its development into a clinically applicable model based on real-world datasets.

To improve the generalizability and clinical relevance of our model, we plan to incorporate real-world data through prospective clinical studies involving diverse patient populations. Such efforts may further refine the model’s robustness and support its clinical applicability.

#### Privacy Protection and Ethical Data Handling

The development of a panic attack prediction model utilizing wearable devices prioritizes the protection of sensitive personal information and adherence to ethical data processing standards. To address these considerations, this study proposes the following approaches.

First, participants are required to undergo an explicit consent process during initial registration. This process includes a clear explanation of how personal data will be collected, stored, and utilized, as well as detailed information about the types of data collected (eg, ECG, HRV, survey data), the purposes of use, and third-party access.

Such transparent procedures aim to build user trust and ensure that personal data are securely and ethically handled, thereby enhancing the reliability of wearable-based health care services.

Additionally, the protection of health care data collected via wearable devices emphasizes the use of real-time encryption, anonymization, and pseudonymization technologies. Pseudonymized data can reduce personal identification risks while maintaining analytical flexibility, thereby safeguarding against external threats [23].

Compliance with international regulations such as the Health Insurance Portability and Accountability Act and the implementation of encryption protocols such as Transport Layer Security/Secure Sockets Layer for transmission and AES-256 for storage are proposed to mitigate sensitive information leakage and enhance data security.

Finally, real-time encryption technologies, such as Data Encryption Standard and Triple Data Encryption Standard, have been shown to be effective in protecting medical data transmitted via wearable devices [28]. These technologies prevent unauthorized access, modification, or misuse of sensitive data, significantly enhancing the security of data transmission.

These approaches provide practical solutions for protecting data privacy and creating a secure data utilization environment for wearable-based health care services. By adhering to ethical standards, this study aims to enhance the reliability and practicality of the panic attack prediction model.

### Multimodal Panic Attack Detection Model Using Surveys

In a study utilizing the experience sampling method with a smart speaker among 20 participants with mild depression, 93.8% (n=19) participants preferred the graphical user interface (GUI).

About 3.5% (n=1) participants opted for a mixed mode, and 2.7% (n=1) participants favored the voice user interface. The findings also indicated that participants generally favored the GUI but tended to select the voice user interface in situations where they were physically occupied [29]. Based on these findings, we propose a panic disorder prediction model that integrates the Panic Disorder Severity Scale (PDSS) survey [30] with the diagnostic criteria from the DSM-IV [31] a standard diagnostic tool published by the American Psychiatric Association.

The *Diagnostic and Statistical Manual of Mental Disorders, 4th Edition* includes criteria for clinical disorders, personality disorders, and general medical conditions and is used for diagnosis by clinical professionals. The Panic Disorder Severity Scale consists of 7 items that evaluate and monitor the severity of panic disorder based on specific scores. By combining these tools, the proposed model not only predicts the likelihood of panic attacks through machine learning but also enables qualitative assessments of psychological states, thereby enhancing the reliability of the predictions.

This model is envisioned as a multimodal tool combining GUI-based surveys with a prediction model. When users begin the application, they first complete a GUI-based survey. These results will then inform the subsequent prediction of panic disorder, leading to more accurate and reliable diagnostic outcomes.

### Conclusions

This study highlights the potential of wearable devices, specifically ECG and HRV monitoring, in predicting panic attacks and informing future digital health strategies. Using a dataset of 45,152 ECG recordings, our random forest model demonstrated exceptional performance with 99.27% accuracy, 99.69% precision, 98.49% recall, and an F1 score of 99.09%. These results suggest the feasibility of integrating wearable technology into future real-time monitoring systems, offering potential for early risk detection, subject to clinical validation. By combining rigorous data preprocessing strategies, age-specific modeling, and machine learning techniques, this study proposes a machine learning framework with interpretable components and potential scalability, subject to broader evaluation.

## Abbreviations

ECG: Electrocardiogram
GUI: graphical user interface
HRV: heart rate variability
IRB: Institutional Review Board
MSE: mean squared error
RF: random forest
RMSSD: root mean square of successive differences
